# Estrogen receptor-negative/progesterone receptor-positive breast cancer has distinct characteristics and pathologic complete response rate after neoadjuvant chemotherapy

**DOI:** 10.1186/s13000-023-01433-6

**Published:** 2024-01-04

**Authors:** He Dou, Fucheng Li, Youyu Wang, Xingyan Chen, Pingyang Yu, Siyuan Jia, Yuling Ba, Danli Luo, Tian Gao, Zhaoting Li, Min Xiao

**Affiliations:** Department of Breast Surgery, Harbin Medical University Cancer Hospital, No.150, Haping Road, Nangang District, Harbin, 150081 Heilongjiang People’s Republic of China

**Keywords:** Breast cancer, Neoadjuvant chemotherapy, Pathologic complete response, Estrogen receptor, Progesterone receptor

## Abstract

**Purpose:**

The status of hormone receptors (HR) is an independent factor affecting survival and chemotherapy sensitivity in breast cancer (BC) patients, with estrogen receptor (ER) and progesterone receptor (PR) having the most significant effects. The ER-/PR + phenotype has been controversial in BC, and experts will face many challenges in determining treatment strategies. Herein, we systematically analyzed the clinicopathological characteristics of the ER-/PR + phenotype in BC patients and the response to chemotherapy.

**Patients and methods:**

We included two cohorts. The first cohort counted the relationship between clinicopathologic data and survival outcomes for 72,666 female patients in the Surveillance, Epidemiology, and End Results (SEER) database. The second cohort analyzed the relationship between clinicopathological data and pathologic complete response (pCR) rate in 879 patients at the Harbin Medical University Cancer Hospital. The classification data were compared by the chi-square test and Fister's exact test of the Logistic regression model, and predictor variables with *P* < 0.05 in the univariate analysis were included in the multivariate regression analysis. The Kaplan–Meier method evaluated breast cancer-specific survival (BCSS) and overall survival (OS) to investigate the relationship between different HR typing and survival and pCR.

**Results:**

In the two cohorts, 704 (0.9%) and 11 (1.3%) patients had the ER-/PR + phenotype, respectively. The clinicopathologic features of patients with the ER-/PR + phenotype are more similar to those of the ER-/PR- phenotype. The ER-/PR + phenotype is more common in younger and premenopausal women, and most ER-/PR + phenotypes exhibit higher histological grades. Survival analysis showed that there were significant differences in OS and BCSS among patients with different HR states (*P* < 0.001). The survival results of patients with the ER + /PR + phenotype were the best. The prognosis of the ER-/PR + phenotype was similar to that of the ER-/PR- phenotype. On the other hand, we found that HR status was also an independent predictor of post-NAC pCR rate in BC patients. The ER + /PR- and ER-/PR- phenotypes were more sensitive to chemotherapy than the ER + /PR + phenotypes.

**Conclusion:**

HR status is the main factor affecting BC's survival outcome and pCR rate. Patients with the ER-/PR + phenotype possess more aggressive biological factors and can benefit significantly from chemotherapy. We need to pay more attention to this group and achieve individualized treatment, which will help us treat BC better and provide new targets and blueprints for our clinical treatment.

## Introduction

Since 2000, cancer incidence has increased significantly worldwide, and 9 million cases died from cancer worldwide in 2020. How to correctly respond to the rapidly increasing burden of cancer is a massive challenge for every country [1]. By the end of 2020, as many as 2.3 million people had been diagnosed with BC worldwide, up 126,000 from 2019, surpassing lung cancer as the most significant number of new cases in the world [2]. The incidence and mortality of BC in China are also increasing yearly. For the treatment of BC, we should combine the expansion of the scope of BC screening with the standardization of clinical treatment. Although the OS rate of BC in China increased by over ten percentage points compared with ten years ago, there is still a big gap with developed countries [3].

BC is now being treated in increasingly diverse ways. Endocrine therapy is much milder and more acceptable to patients than several other treatments. It is well known that the primary mechanism of endocrine therapy is the regulation of reproductive hormones in the body, which play a crucial role in BC, and women with a high average distribution of hormone levels have two to three times higher risk of BC than average women [4]. Reproductive hormones were found to change before and after neoadjuvant chemotherapy (NAC), and the treatment regimen can be appropriately adjusted according to the changes in these hormones [5]. Most BC shows hormone-dependent growth, mainly manifested as the combination of estrogen, progesterone, and the receptor on the surface of tumor cells, thus stimulating tumor growth. Anti-hormone therapy can control the growth of tumor cells and kill tumor cells [6].

The expression of HR has a significant predictive value for endocrine therapy effects [7]. BC can be typed according to the HR status: ER + /PR + , ER + /PR-, ER-/PR + , ER-/PR-. It has been found that patients can benefit from endocrine therapy when both ER and PR are highly expressed and have higher survival rates compared to other phenotypes [8]. Thakkar found that the ER + /PR- phenotype is a unique subset of BC, classified as luminal B tumors characterized by an invasive nature. The loss of this phenotype PR expression also demonstrates the abnormal function of ER, resulting in tamoxifen resistance [9, 10]. However, patients with the ER-/PR- phenotype have a high overall risk of recurrence and a short survival cycle, which can significantly benefit from chemotherapy and have a low response to endocrine therapy. However, the ER-/PR + phenotype is still controversial, and whether it is an artifact or an actual phenotype still needs further investigation. American Society of Clinical Oncology (ASCO) recommends that clinicians perform repeat testing after finding an ER-/PR + phenotype to avoid false-negative ER findings [11]. Some experts believe that a technical error causes the ER-/PR + phenotype. The pathology of BC patients requires immunohistochemistry (IHC) to evaluate the expression of ER, PR, human epidermal growth factor receptor 2 (HER-2), and Ki67 [12]. Tissue fixation or excessive staining time may weaken the ability of IHC antibodies to detect staining [13]. An additional group of experts found that PR is not necessarily downregulated when the expression level or level of ER is reduced, suggesting that PR regulation is independent of ER so that the ER-/PR + phenotype can be present [14]. Therefore, this study evaluated the clinicopathological characteristics of patients with the ER-/PR + phenotype and the pCR rate after NAC compared to BC patients with other phenotypes (ER + /PR + , ER + /PR-, ER-/PR-).

## Material and methods

### Two study cohorts

Cohort 1 from the SEER database in the United States, the SEER database (http://seer.cancer.gov/) is the most extensive population-based publicly available cancer data set, including 18 population-based cancer registries in 14 states, sponsored by the National Cancer Institute and covering about 26% of the US population, included tumor incidence and subsequent survival of the population. Our team applied for access permission on the official website and logged in through the official account (user name: 24,753-Nov2021) using the SEER *STat version 8.4.0.1 (http://seer.cancer.gov/seerstat) provided by the National Cancer Institute. We selected female patients diagnosed with BC between 1 January 2010 and 31 December 2015, which allowed a better follow-up time and made the results more convincing, with 102,008 patients screened. Meanwhile, we retrieved the year and age of confirmed BC, race, differentiation grade, T stage, N stage, M stage, stage, ER status, PR status, HER-2 status, histological type, survival time, and cause of death.

**Fig. 1:**
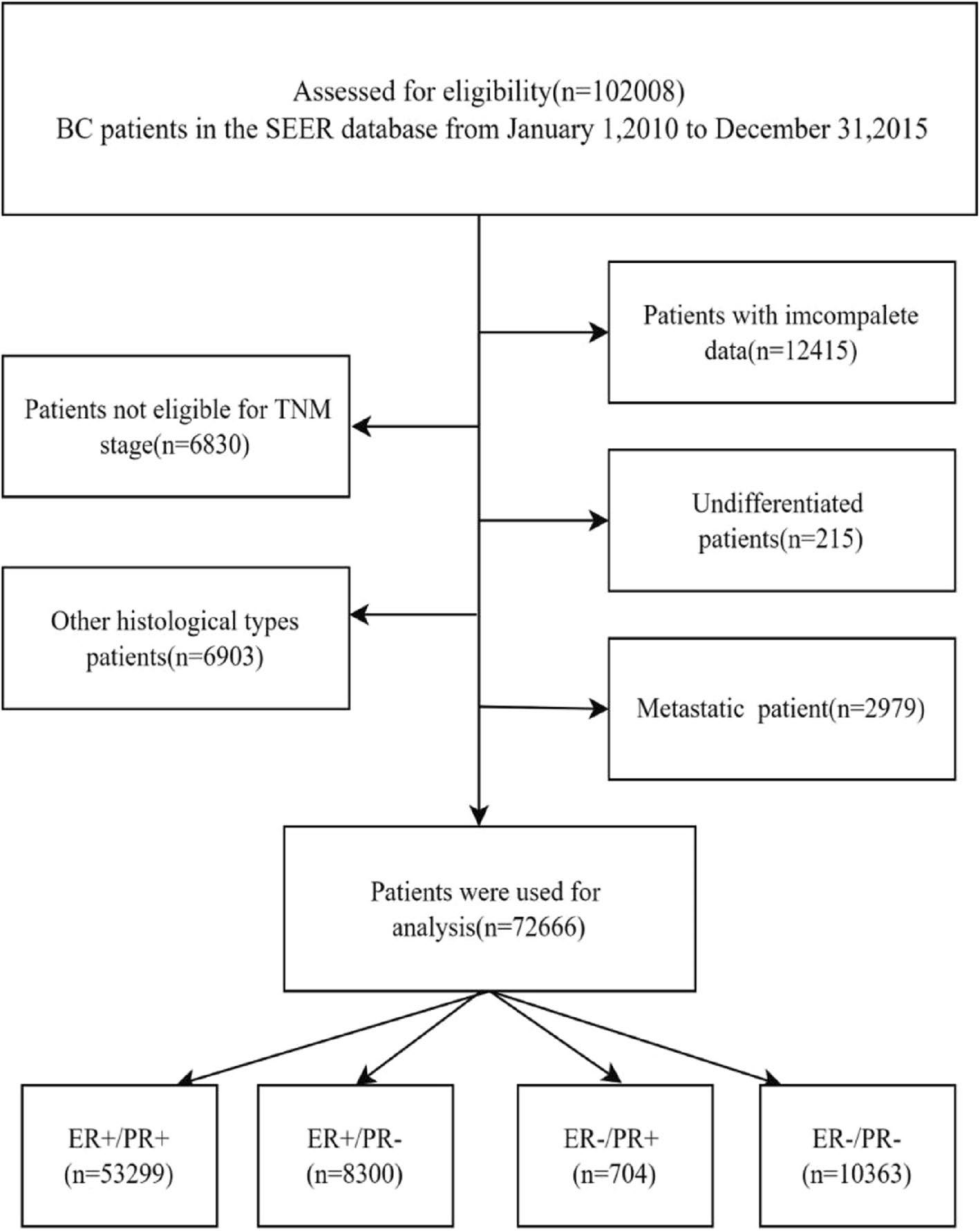
Grouping flow chart of 72,666 BC patients collected in cohort 1

Our team screened the 102,008 patients based on the following criteria. Specific inclusion criteria are as follows: (1) determine cancer site and histological type reference International Classification of Disease for Oncology third edition ICD-O-3(https://www.naaccr.org/icdo3/), and use the ICD-O-3 codes C500 to C506, C508 to C509 to select BC; (2) select invasive ductal carcinoma (IDC) or invasive lobular carcinoma (ILC), the codes are 8500/3,8520/3,8521/3,8522/ 3,8524/3 and 8541/3; (3) select patients with T1-T4 and N0-N3 tumors in the American Joint Committee on Cancer (AJCC) TNM staging system; (4) the age range is from 20–80 years old; (5) the patient did not develop distant metastasis. The exclusion criteria were as follows: (1) patients with incomplete data; (2) patients with unknown causes of death; (3) patients with multiple tumors or distant metastases; (4) patients with other histological types. The specific screening procedure is described in Fig. 1, with 72,666 patients entering the study. Details of the patients are given in Table 1.

**Table 1:** The characteristics of patients BC in different HR status in Cohort 1

Patient characteristic		**HR status**				*P*
		ER + /PR +	ER + /PR-	ER-/PR +	ER-/PR-	
		N (%)	N (%)	N (%)	N (%)	
Total		53,299 (73.4)	8300 (11.4)	704 (0.9)	10,363 (14.3)	
Age at diagnosis	20–30	220 (0.4)	62 (0.7)	8 (1.1)	130 (1.3)	** < 0.001**
	31–40	2014 (3.7)	381 (4.6)	67 (9.5)	870 (8.4)	
	41–50	9410 (17.7)	950 (11.4)	165 (23.4)	1940 (18.7)	
	51–60	13,711 (25.7)	2331 (28.1)	186 (26.4)	3034 (29.3)	
	61–70	16,733 (31.4)	2781 (33.5)	169 (24.0)	2829 (27.3)	
	71–80	11,211 (21.1)	1795 (21.7)	109 (15.6)	1560 (15.0)	
Race	Black	3540 (6.6)	997 (12.0)	93 (13.2)	1553 (15.0)	** < 0.001**
	White	42,977 (80.6)	6255 (75.4)	535 (76.0)	7459 (72.0)	
	Others	6782 (12.8)	1048 (12.6)	76 (9.8)	1351 (13.0)	
Histological grade	1	16,293 (3.6)	1509 (18.2)	11 (1.6)	159 (1.5)	** < 0.001**
	2	27,008 (50.7)	3424 (41.3)	127 (18.4)	1919 (18.5)	
	3	9998 (46.7)	3367 (40.5)	566 (80.0)	8285 (80.0)	
T stage	1	36,432 (68.4)	4965 (59.8)	304 (43.2)	5022 (48.5)	** < 0.001**
	2	13,641 (25.6)	2600 (31.3)	322 (45.7)	4090 (39.5)	
	3	2536 (4.8)	554 (6.7)	52 (7.4)	840 (8.1)	
	4	690 (1.2)	181 (2.2)	26 (3.7)	411 (3.90)	
N stage	0	40,502 (76.0)	6083 (73.3)	480 (68.2)	7058 (68.1)	** < 0.001**
	1	9385 (17.6)	1543 (18.6)	164 (23.2)	2336 (22.5)	
	2	2319 (4.4)	405 (4.9)	37 (5.3)	556 (5.4)	
	3	1093 (2.0)	269 (3.2)	23 (3.3)	413 (4.0)	
HER-2	Positive	5311 (9.9)	1909 (23.0)	197 (28.0)	2966 (28.6)	** < 0.001**
	Negative	47,988 (90.1)	6391 (77.0)	507 (72.0)	7397 (71.4)	
Stage	I	31,842 (59.7)	4314 (52.0)	252 (35.8)	4193 (40.5)	** < 0.001**
	II	16,643 (31.2)	2994 (36.1)	358 (50.8)	4561 (44.0)	
	III	4814 (9.1)	992 (11.9)	94 (13.4)	1609 (15.5)	
Histological type	IDC	42,621 (80.0)	6697 (80.7)	671 (95.3)	10,035 (96.8)	** < 0.001**
	ILC	6422 (12.0)	1055 (12.7)	13 (1.8)	123 (1.2)	
	Others	4256 (8.0)	548 (6.6)	20 (2.9)	205 (2.0)	

*Abbreviation*: *HR* Hormone receptor, *ER* Estrogen receptor, *PR* Progesterone receptor, *HER-2* Human epidermal growth factor receptor 2, *IDC* Invasive ductal carcinoma, *ILC* Invasive lobular carcinoma. Bold values indicate that they are statistically significant at *P* ≤ 0.05

In Cohort 2, from Harbin Medical University Cancer Hospital, we selected 1424 patients treated in our hospital from 1 January 2012 to 31 December 2019 who underwent hollow needle biopsy to confirm BC before treatment. Patients underwent chemotherapy according to the standard guidelines, and the chemotherapy cycle was complete; surgical treatment after complete NAC, surgical mode with mastectomy or breast-conserving surgery (BCS) according to the patient's disease condition and intention, all patients underwent sentinel lymph node biopsy (SLNB), axillary lymph node dissection (ALND) if SLNB node metastasis.

**Fig. 2:**
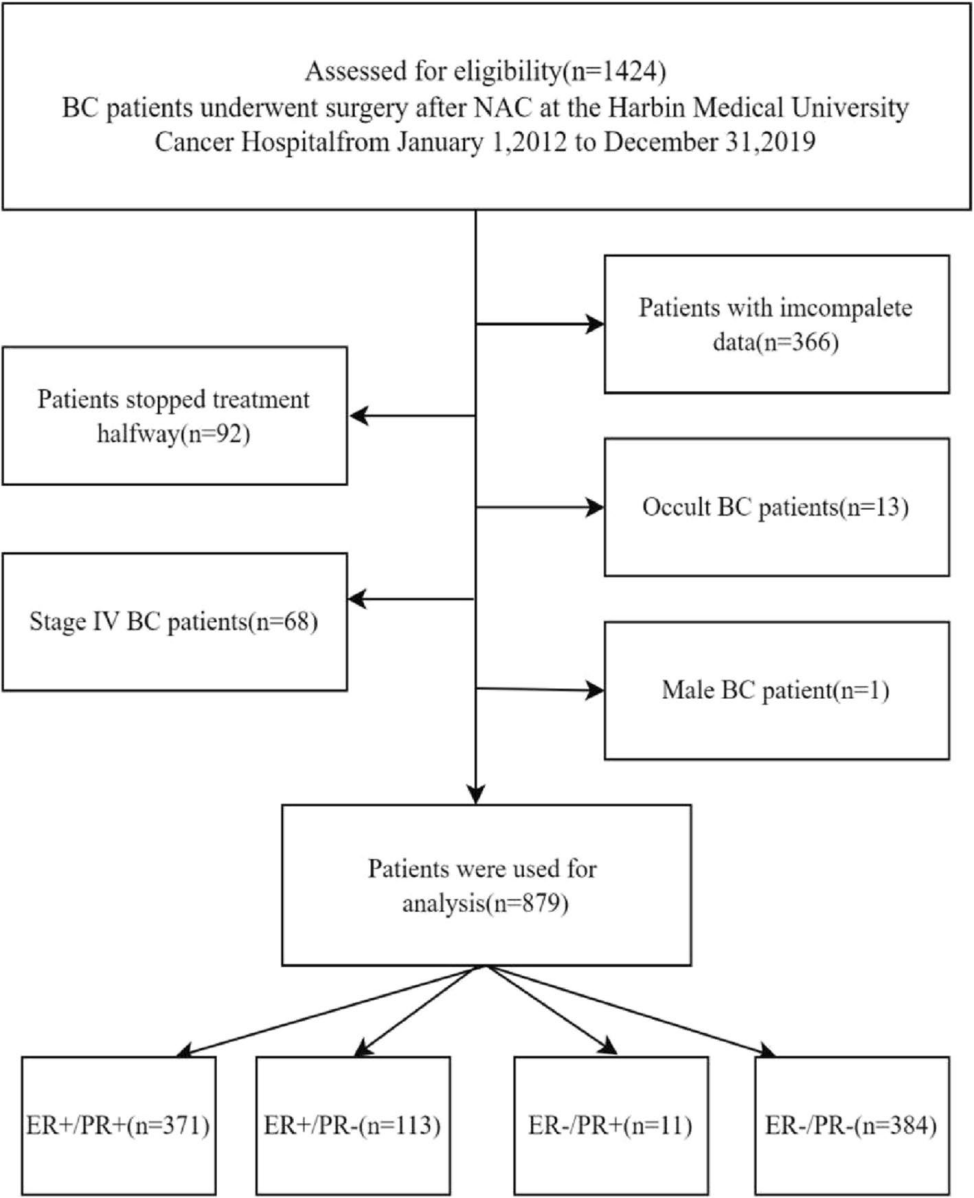
Grouping flow chart od 879 BC patients collected in cohort 2

A total of 1424 patients were obtained for analysis, and detailed inclusion criteria included: (1) female patients; (2) pathological confirmation of BC before chemotherapy; (3) all patients received NAC and completed treatment; (4) complete clinical and pathological data; (5) T1-T3 tumors specified in the TNM staging system of AJCC; (6) patients underwent pathological IHC testing at the beginning and end of NAC. Exclusion criteria included: (1) patients with incomplete data; (2) patients with multiple tumors; (3) patients lacking age at diagnosis and life status; (4) patients with occult BC; (5) male patients; (6) patients with interrupted treatment or treatment in other hospitals. Finally, 879 patients who met this index were selected for the analysis. This flow chart is shown in Fig. 2. The baseline characteristics of this cohort in the present study are shown in Table 2.

**Table 2:** The characteristics of patients BC in different HR status in Cohort 2

Patient characteristic		**HR status**				*P*
		ER + /PR +	ER + /PR-	ER-/PR +	ER-/PR-	
		N (%)	N (%)	N (%)	N (%)	
Total		371 (42.2)	113 (12.9)	11 (1.3)	384 (56.4)	
Age at diagnosis	20–40	74 (19.9)	19 (16.8)	4 (36.4)	63 (16.4)	0.533
	41–60	257 (69.3)	81 (71.7)	7 (63.6)	276 (71.9)	
	61–80	40 (10.8)	13 (1.5)	0 (0)	45 (11.7)	
Surgical methods	BCS	21 (5.7)	4 (3.5)	1 (9.0)	10 (2.6)	0.153
	M	350 (94.3)	109 (96.5)	10 (91.0)	374 (97.4)	
Menstruatio	Yes	137 (36.9)	70 (61.9)	1 (9.0)	212 (55.2)	** < 0.001**
	No	234 (63.1)	43 (38.1)	10 (91.0)	172 (44.8)	
BMI	≤ 18.5	6 (1.6)	4 (3.5)	0 (0)	9 (2.3)	0.090
	18.5–24	160 (43.1)	48 (42.5)	8 (72.7)	182 (47.4)	
	24–30	183 (49.3)	58 (48.7)	1 (9.1)	167 (43.5)	
	≥ 30	22 (6.0)	3 (2.7)	2 (18.2)	26 (6.8)	
Clinical T stage	1	45 (12.1)	17(15.0)	1 (9.1)	44 (11.4)	0.588
	2	277 (74.6)	81 (71.6)	8 (72.7)	271 (70.5)	
	3	49 (13.3)	15 (13.4)	2 (18.2)	69 (18.1)	
Clinical N stage	0	52 (14.0)	11 (9.7)	2 (18.1)	45 (11.7)	0.373
	1	218 (58.7)	62 (54.8)	7 (63.8)	212 (55.2)	
	2	41 (11.2)	18 (16.1)	0 (0)	41 (10.6)	
	3	60 (16.1)	22 (19.4)	2 (18.1)	86 (22.5)	
HER-2	Positive	70 (18.9)	43 (38.1)	3 (37.3)	218 (56.8)	** < 0.001**
	Negative	301 (81.1)	70 (61.9)	8 (62.7)	166 (43.2)	
KI67	≤ 15	171 (46.1)	36 (29.2)	3 (37.3)	101 (26.3)	** < 0.001**
	> 15	200 (53.9)	77 (70.8)	8 (62.7)	283 (73.7)	
P53	0	209 (56.3)	57 (50.4)	4 (36.3)	191 (49.7)	** < 0.001**
	1	123 (33.1)	34 (30.0)	4 (36.3)	48 (12.5)	
	2	24 (6.6)	12 (10.8)	1 (9.0)	59 (15.3)	
	3	15 (4.0)	10 (8.8)	2 (18.4)	86 (22.5)	
Histological grade	0–1	109 (29.4)	39 (34.5)	3 (37.3)	175 (45.6)	** < 0.001**
	2	250 (67.4)	65 (57.5)	6 (44.6)	130 (33.9)	
	3	12 (3.2)	9 (8.0)	2 (18.1)	79 (20.5)	
Stage	I	7 (1.8)	0 (0)	0 (0)	6 (1.6)	0.359
	II	238 (64.2)	67 (59.3)	7 (63.6)	220 (58.4)	
	III	126 (34.0)	46 (40.7)	4 (36.4)	158(40.0)	
Histological type	IDC	279 (75.2)	75 (66.4)	9 (82.0)	219 (57.0)	** < 0.001**
	ILC	43 (11.6)	15 (13.3)	1 (9.0)	53 (13.8)	
	Others	49 (12.2)	23 (20.3)	1 (9.0)	112 (29.2)	

*Abbreviation*: *HR* Hormone receptor, *ER* Estrogen receptor, *PR* Progesterone receptor, *HER-2* Human epidermal growth factor receptor 2, *IDC* Invasive ductal carcinoma, *ILC* Invasive lobular carcinoma, *BCS* Breast conserving surgery, *M* Mastectomy. Bold values indicate that they are statistically significant at *P* ≤ 0.05

This research complies with the World Medical Association Declaration of Helsinki in 1964 and subsequently amended versions. All of the patients signed an informed consent form before the treatment.

### Clinical and pathological variables

In the study variables of cohort 1, the age range was 20–30 years old, 31–40 years old, 41–50 years old, 50–60 years old, and over 60 years old. Races are divided into black, white, and other. According to the degree of epithelial duct formation, nuclear pleomorphism, and nucleoside count, the histological grades were classified as I (Well differentiated), II (Moderately differentiated), and III (Poorly differentiated). The status of patient HR was tested by IHC. ER, and PR positivity was defined as 1% nuclear staining of tumor cells. HER-2 was positive for IHC staining 3 + , 0 or 1 + HER-2 negative for IHC staining, and when IHC staining 2 + , its status was detected by fluorescence in situ hybridization (FISH). HER-2 was considered negative when Fish was negative. Otherwise, it is a positive one. The endpoints for this cohort were.

BCSS and overall OS. BCSS is defined as the time from the date of diagnosis to death due to BC, and OS is defined as the time of death from any cause.

Study variables in cohort 2 included patient age, surgical procedure, menopausal status, body mass index (BMI) values, ER status, PR status, HER-2 status, KI67 expression, P53 expression, T stage, N stage, pathology type, histological grade, and pCR status. Patient information and treatment details were recorded from the beginning of the diagnosis.

The surgical procedure was divided into mastectomy and BCS. Natural Menopause was defined as future menstruation over 12 months or older than 60 years. BMI values were stratified according to International Health Standards: lean, BMI < 18.5; normal, 18.5 ≤ BMI < 24; overweight, 24 ≤ BMI < 30, and obese BMI ≥ 30. The ER, PR, and HER-2 status in cohort 2 were also detected with IHC. Ki67 refers to the anti-reproductive protein monoclonal antibody, a hyperplastic cell nuclear antigen.

associated with the tumor cell cycle, which is interpreted as the percentage of tumor cell nuclei between 400 and 500 cells.15% of KI67 positive nuclei were high and < 15% were low. All patients underwent clinical and radiographic staging. T stage was determined by palpation and ancillary examination methods. N stage was defined as axillary lymph nodes or ultrasound-detected lymph node abnormalities. Metastatic disease was assessed by imaging examination. We classified the patients into four types according to their ER and PR status: ER + /PR + , ER + /PR-, ER-/PR + , and ER-/ PR-. Pathologists observed tumor sections and analyzed pathological types, such as IDC, ILC, etc. According to the pathological assessment after the NAC of the Chinese Society of Clinical Oncology, the current evaluation of the primary lesions using the Miller & Payne system. This system mainly compares the pre- and post-treatment surgical specimens. To assess the abundance of residual infiltrating tumor cells after NAC, specific interpretation criteria are divided into the following five levels: Grade 1 (G1): no change in invasive cancer cells or only a single cancer cell, But the total number of cancer cells did not decrease; Grade 2 (G2): mild reduction of invasive cancer cells, But the total number is still relatively high, No more than 30% of the number of cancer cells; Grade 3 (G3): means the reduction of invasive cancer cells by 30% to 90%; Grade 4 (G4): the cancer cell infiltration rate reaches more than 90%, Only a small number of scattered cancer cells or a single cancer cell; Grade 5 (G5): refers to the absence of invasive cancer cells in the original tumor bed site, But ductal carcinoma in situ. G5 was used here as the study endpoint for cohort 2.

### Statistical analysis

Data presented in this paper use the SPSS software version 26.0 (IBM Corporation, New York, USA) to analyze and stratify the data in this paper. Categorical data are expressed as counts and percentages by molecular typing, primary data of patients were continued when compared and analyzed using the chi-square test, and correlations between clinical case parameters and BCSS, OS, or pCR rate within each subgroup were performed using the chi-square test and the univariate Logistic regression analysis. Statistically, significant variables from the univariate analysis were included in the multivariate analysis. To determine which variables are independent of BCSS, OS, or pCR rate. Survival curves were generated using the Kaplan–Meier method, and the log-rank test was used to assess the survival differences between the groups. The Cox proportional hazards model calculated multivariate-adjusted hazard ratios with 95% confidence intervals (CI). *P* < 0.05 was considered statistically significant.

## Results

### Characteristics of study sample

In cohort 1, we screened 72,666 eligible female patients from the SEER database, ranging in age from 20 to 80 years old, and most of the patients (30.9%) were between 61 and 70 years old. The HR status was closely related to age, race, histological grade, T stage, N stage, HER-2 status, stage, and histological type (*P* < 0.001). The histological type of most patients was IDC (82.6%, n = 60,024). 73.5% of patients in the entire cohort were ER + /PR + phenotype, 10,363 (14.3%) patients were ER-/PR- phenotype, 8300 (11.4%) patients were ER + /PR- phenotype, and only 704 (0.9%) patients were the ER-/PR + phenotype. Compared with the ER + /PR + and ER + /PR- phenotypes, patients with ER-/PR + phenotypes had relatively higher histological grades. Most patients, up to 80%, were grade III. The proportion of patients with positive HER-2 was relatively lower and had larger tumor sizes and higher lymph node metastasis rates (*P* < 0.001). The overall clinicopathological features of patients with the ER-/PR + phenotype were similar to those of the ER-/PR- phenotype.

In cohort 2, we mainly studied 1424 patients diagnosed as BC and received NAC in the Harbin Medical University Cancer Hospital from January 1, 2012, to December 31, 2019. excluded 545 patients (366 patients without complete data, 92 patients stopped treatment or transferred to another hospital, 13 patients diagnosed with hidden BC, 1 male patient, 68 stage IV BC), a total of 879 patients were included in this study, the age range of 21–72 years, the median age of 52 years. Different HR status was closely related to menopausal status, HER-2 status, KI67 expression, P53 expression, histological grade, and histological type (*P* < 0.05). In this cohort, younger patients were more likely to receive BCS, but the overall breast conservation rate was not high, only 4.1%, which may be related to the treatment environment and the mentality of the patients at that time. 11 (1.3%) patients were diagnosed with an ER-/PR + phenotype in a proportion similar to cohort 1. Most patients were young and unmenopausal. Biomarkers such as KI67, P53, and HER-2 are highly expressed in the ER-/PR + phenotype. The observed differences in HR status versus tumor size in cohort 1 were not certified in cohort 2, which is most likely responsible for insufficient patients. The majority of patients (66.3%) had IDC, while the remaining patients had ILC (2.4%) and other types of cancer (31.4%).

### Survival outcomes of BC with ER-/PR + phenotype in Cohort 1

**Fig. 3:**
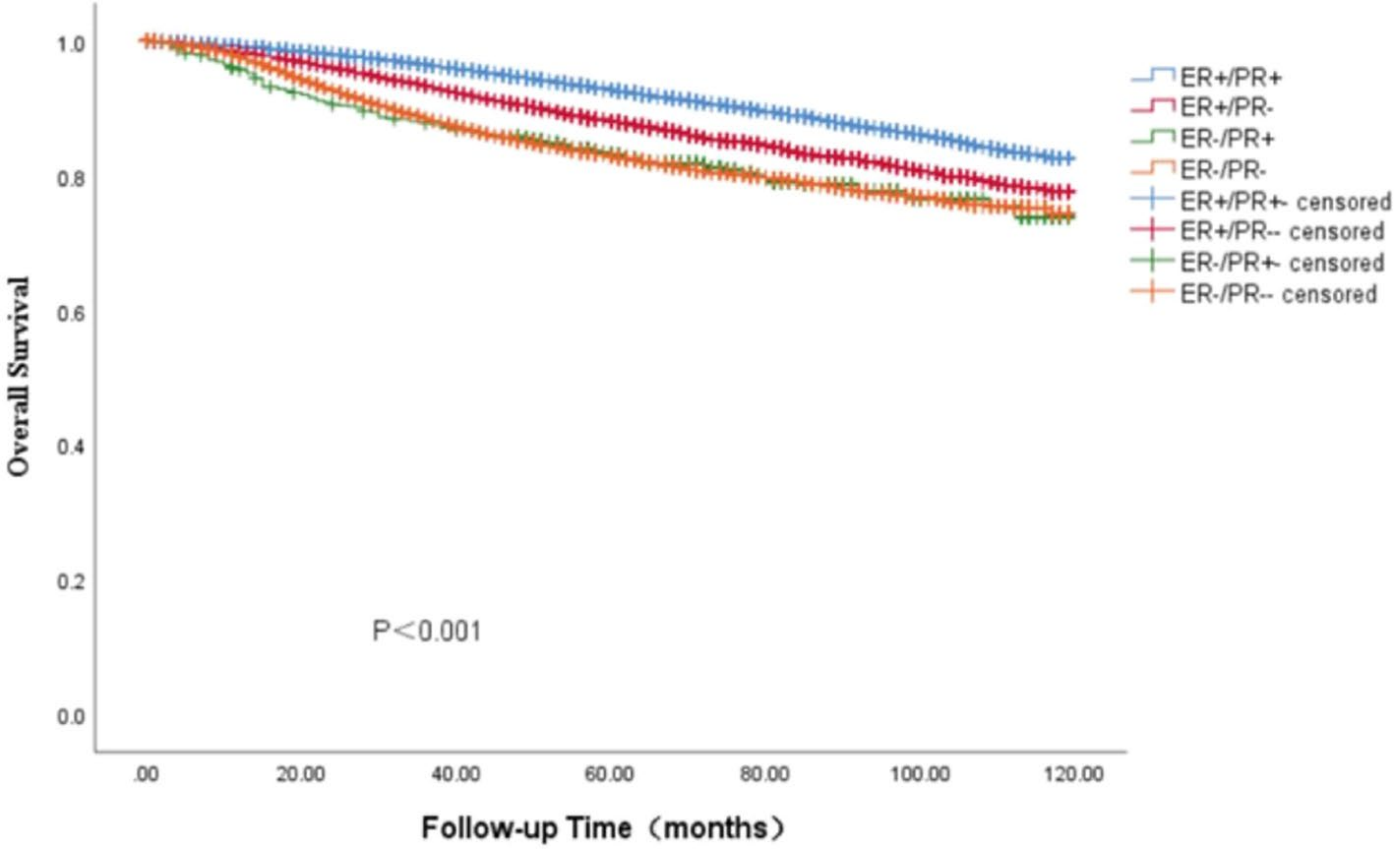
Relationship of different HR combination subgroups and OS in the whole series (Kaplan–Meier method and log-rank test) Legend: The four different HR combinations were associated with significant differences in patient OS (log-rank, *P* < 0.001). The prognosis of patients with the ER-/PR + phenotype was similar to that of patients with the ER-/PR- phenotype (*P* = 0.978), and patients with the ER + /PR + and ER + /PR- phenotypes had a significantly better survival outcome than the other patients (*P* < 0.001), patients with ER-/PR + phenotype had a higher risk of death than other patients, and the mean survival time was 100.45 months. Abbreviation: ER, estrogen receptor; PR, progesterone receptor

**Fig. 4:**
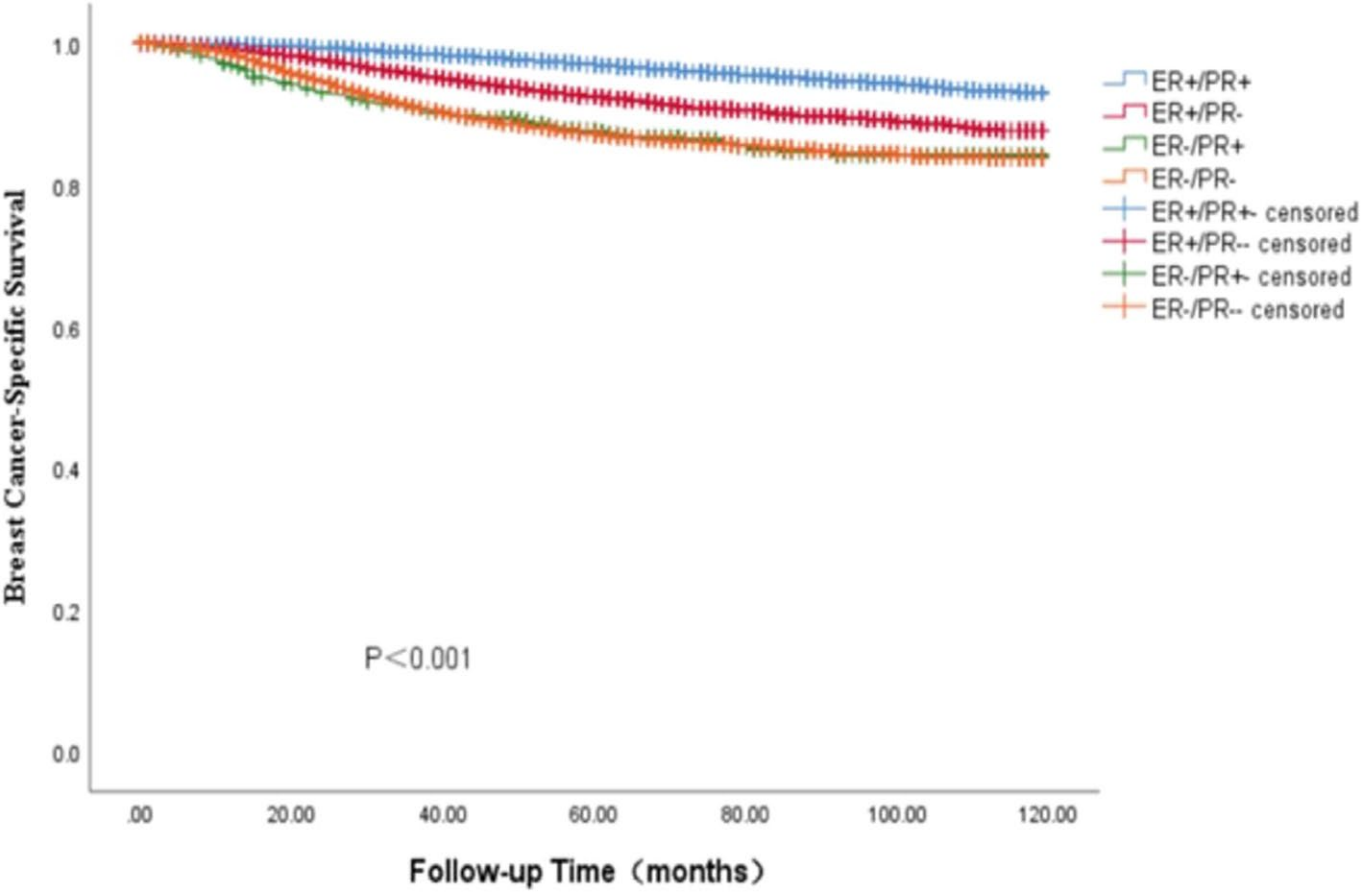
Relationship of different HR combination subgroups and BCSS in the whole series (Kaplan–Meier method and log-rank test) Legend: The four different HR combinations were significantly different from the BCSS of the patients (log-rank, *P* < 0.001). The prognosis of the patients with the ER-/PR + phenotype was similar to that of the patients with the ER-/PR- phenotype (*P* = 0.995), the best prognosis of the patients with the ER + /PR + phenotype, and the prognosis of the patients with the ER + /PR- phenotype was situated between the ER + /PR + phenotype (best outcome) and ER-/PR + phenotype (worst outcome) (*P* < 0.001). Patients with ER-/PR- phenotype had a higher risk of death than the other groups, with a mean survival time of 106.17 months. Abbreviation: ER, estrogen receptor; PR, progesterone receptor

**Fig. 5:**
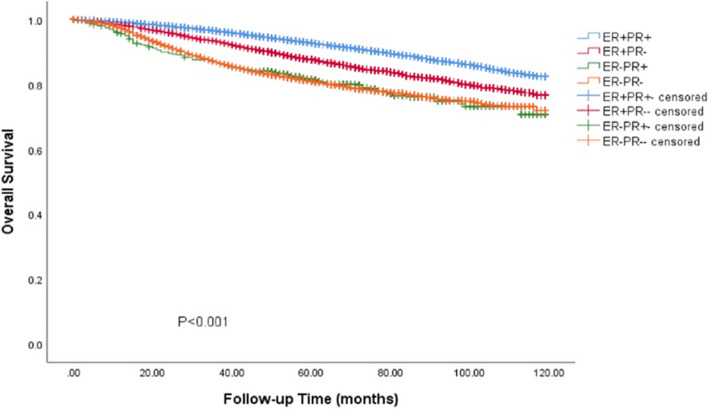
Relationship of different HR combination subgroups and OS in the HER-2 negative series (Kaplan–Meier method and log-rank test) Legend: The four different HR combinations were significantly different from the OS of the HER-2 negative patients (log-rank,* P* < 0.001). Among HER-2-negative patients, patients with ER-/PR + phenotype had the worst prognosis, but it was not significantly different from that of patients with ER-/PR- phenotype (*P* = 0.899), and patients with ER + /PR + phenotype had the best prognosis and the lowest risk. The prognosis of patients with ER + /PR- phenotype was located in the middle of the ER + /PR + phenotype (the best outcome) and ER-/PR + phenotype (the worst outcome) between them (*P* < 0.001). Abbreviation: ER, estrogen receptor; PR, progesterone receptor; HER-2, human epidermal growth factor receptor 2

**Fig. 6:**
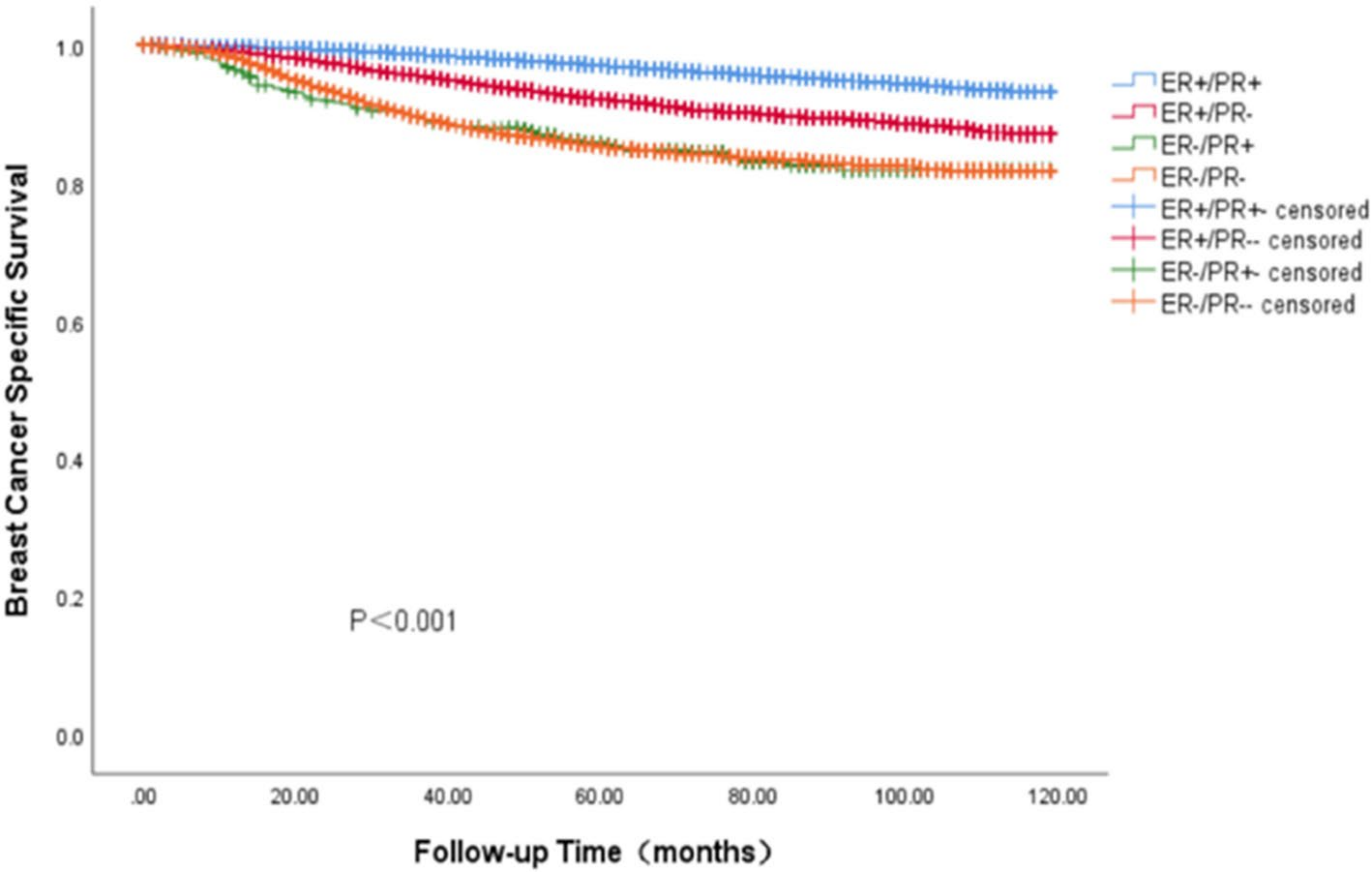
Relationship of different HR combination subgroups and BCSS in the HER-2 negative series (Kaplan–Meier method and log-rank test) Legend: The four different HR combinations were significantly different from the BCSS of the HER-2 negative patients (log-rank,* P* < 0.001). Among HER-2-negative patients, patients with ER-/PR + phenotype had the worst prognosis, but it was not significantly different from that of patients with ER-/PR- phenotype (*P* = 0.918), and patients with ER + /PR + phenotype had the best prognosis and the lowest risk. The prognosis of patients with ER + /PR- phenotype was located in the middle of the ER + /PR + phenotype (the best outcome) and ER-/PR + phenotype (the worst outcome) between them (*P* < 0.001). The overall results were similar to the whole group of patients. Abbreviation: ER, estrogen receptor; PR, progesterone receptor; HER-2, human epidermal growth factor receptor 2

**Fig. 7:**
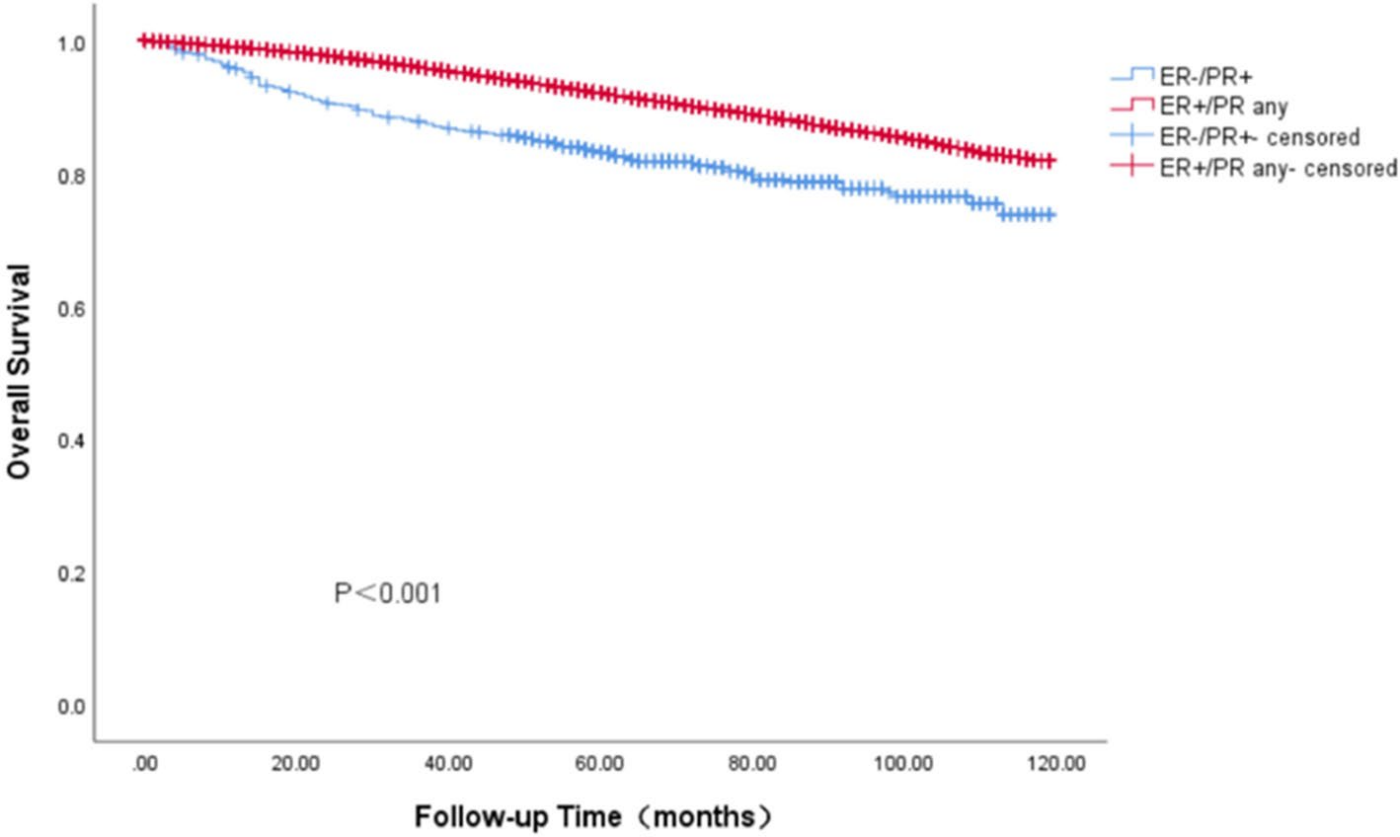
OS relationship between the ER-/PR + and ER + /PR any groups (Kaplan–Meier method and log-rank test) Legend: Regardless of how PR was expressed, when patients were positive for ER expression, their OS was significantly better than that of patients with ER-/PR + phenotypes (log-rank,* P* < 0.001). Abbreviation: ER, estrogen receptor; PR, progesterone receptor

**Fig. 8:**
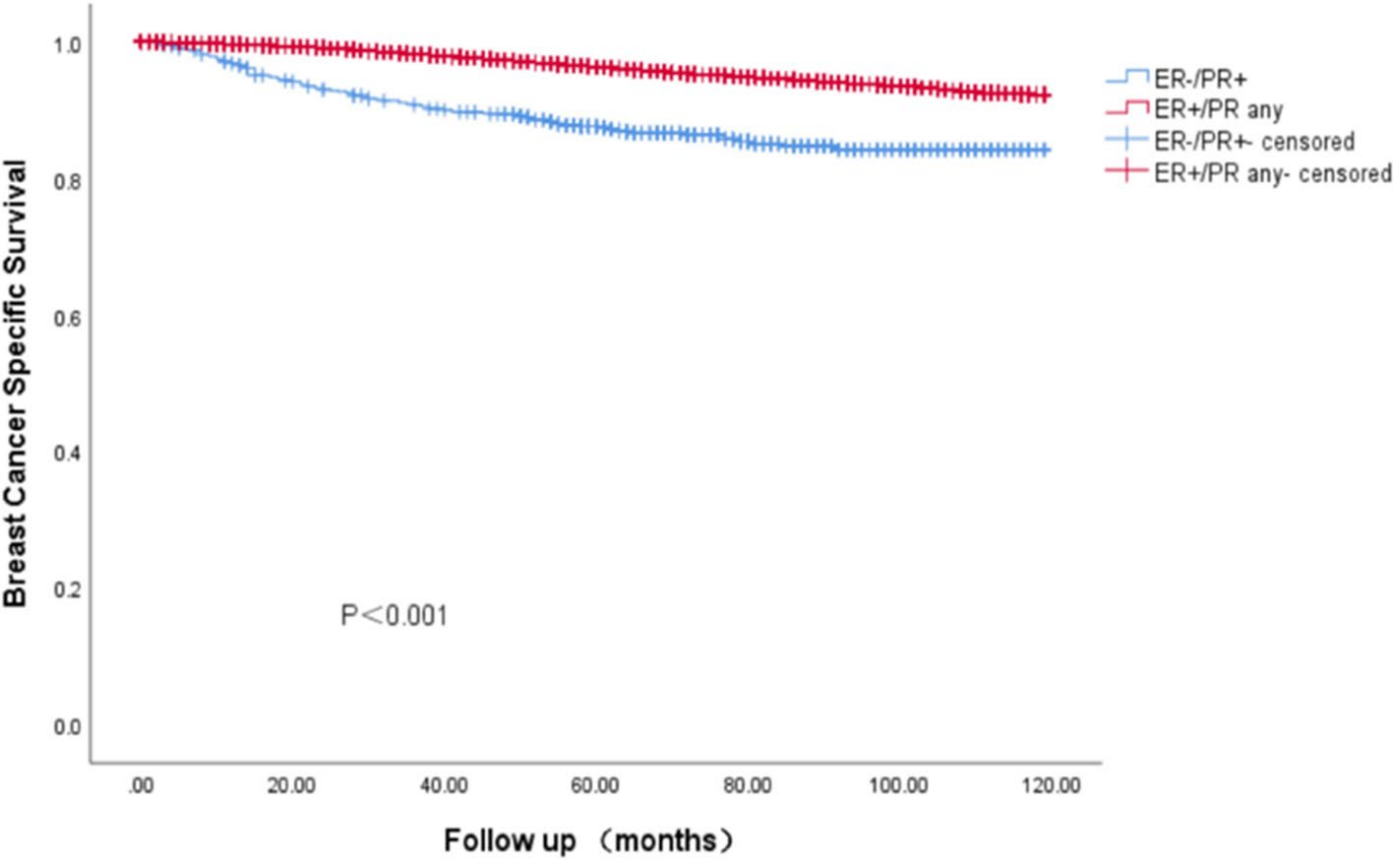
BCSS relationship between the ER-/PR + and ER + /PR any groups (Kaplan–Meier method and log-rank test) Legend: Regardless of how PR was expressed, when patients were positive for ER expression, their BCSS was significantly better than that of patients with ER-/PR + phenotypes (log-rank,* P* < 0.001). Abbreviation: ER, estrogen receptor; PR, progesterone receptor

In the SEER cohort, the survival data analysis showed a median follow-up of 75 months (range: 0—119 months), with the four HR curves being significantly different from OS and BCSS (*P* < 0.001). We found that the ER + /PR + phenotype had the best prognosis, with 5-year OS and BCSS rates of 92.7% and 96.9%, ER-/PR + and ER-/PR- phenotype having similar survival outcomes, with 5-year OS rates of 88.1% and 82.8%, respectively, and 5-year BCSS rates of 87.4% and 87.1%, The ER + /PR- phenotype between the three subgroups, the 5-year OS and BCSS were 88.1% and 92.4% (Figs. 3 and 4). As HER-2 positivity is a biologically distinct phenotype, we excluded HER-2 positive patients from this cohort. We continued investigating the relationship between different HR statuses with OS and BCSS in HER-2 negative BC patients (Figs. 5 and 6). We found that different HR statuses remained significantly associated with OS and BCSS when patients had negative HER-2 expression (*P* < 0.001). Patients with ER + /PR- phenotype also had higher 5-year OS and BCSS rates (92.7%, and BCSS: 97.0%). The survival outcome for the ER-/PR + phenotype was between the ER + /PR- and ER-/PR- groups (OS:85.8% vs. 87.6% vs. 80.9%, and BCSS:85.8% vs. 92.1% vs. 85.3%). We subsequently compared the survival outcomes of the ER-/PR + phenotype with the ER + /PR any phenotype to determine the effect of ER expression on survival (Figs. 7 and 8). We found that the 5-year OS and BCSS of the ER + /PR any phenotype remained significantly higher than the ER-/PR + phenotype (OS: 92.1% vs. 83.2%, and BCSS: 96.3% vs. 87.4%, *P* < 0.001).

### Univariate and multivariate Cox regression analysis of clinicopathological factors associated with OS and BCSS in Cohort 1

Univariate analysis was used to determine the clinicopathological factors affecting the prognosis of BC patients (Table 3). The results showed that negative ER and PR were the risk factors affecting OS and BCSS and that the risk was higher when ER-negative was expressed. Significant differences between the ER-/PR + phenotype and ER + /PR + survival (OS: Hazard Ratio = 2.036, CI 95% 1.721–2.409, *P* < 0.001; BCSS: Hazard Ratio = 3.435, CI 95% 2.798–4.217, *P* < 0.001). When statistically significant factors in univariate analysis were included in multivariate analysis, we found that age, race, histological grade, T stage, N stage, stage, and HR status were independent influencing factors affecting OS and BCSS (*P* < 0.001) (Table 4). In conclusion, the OS and BCSS of patients are different when HR expression is different. The clinicopathological features and survival outcomes of the ER-/PR + phenotype were lower than the ER + /PR + phenotype and ER + /PR- phenotype and similar to the ER-/PR- phenotype.

**Table 3:** Univariate analysis of the OS and the BCSS in Cohort 1

Patient characteristic		OS			BCSS		
		Hazard Ratio	CI (95%)	*P*	Hazard Ratio	CI (95%)	*P*
Age at diagnosis	20–30	Ref			Ref		
	31–40	0.796	0.611–1.038	0.092	0.859	0.644–1.145	0.300
	41–50	0.482	0.374–0.622	** < 0.001**	0.444	0.336–0.623	** < 0.001**
	51–60	0.592	0.463–0.765	** < 0.001**	0.473	0.359–0.623	** < 0.001**
	61–70	0.744	0.580–0.955	**0.020**	0.396	0.300–0.521	** < 0.001**
	71–80	1.517	1.182–1.947	**0.001**	0.542	0.411–0.714	** < 0.001**
Race	White	Ref			Ref		
	Black	1.728	1.627–1.835	** < 0.001**	2.149	1.984–2.327	** < 0.001**
	Others	0.805	0.752–0863	** < 0.001**	0.931	0.848–1.023	0.136
Histological grade	1	Ref					
	2	1.435	1.351–1.524	** < 0.001**	2.471	2.202–2.772	** < 0.001**
	3	2.342	2.206–2.485	** < 0.001**	6.389	5.721–7.135	** < 0.001**
T stage	1	Ref					
	2	1.994	1.906–2.085	** < 0.001**	3.830	3.573–4.105	** < 0.001**
	3	3.214	3.000–3.442	** < 0.001**	7.738	7.075–8.464	** < 0.001**
	4	7.047	6.467–7.680	** < 0.001**	17.291	15.543–19.236	** < 0.001**
N stage	0	Ref					
	1	1.750	1.666–1.839	** < 0.001**	3.188	2.977–3.414	** < 0.001**
	2	3.018	2.814–3.237	** < 0.001**	6.288	5.759–6.866	** < 0.001**
	3	4.921	4.553–5.318	** < 0.001**	11.135	10.14–12.220	** < 0.001**
ER	Negative	Ref					
	Positive	0.528	0.503–0.553	** < 0.001**	0.337	0.317–0.358	** < 0.001**
PR	Negative	Ref					
	Positive	0.561	0.543–0.591	** < 0.001**	0.361	0.341–0.383	** < 0.001**
HER-2	Negative	Ref					
	Positive	0.990	0.934–1.050	0.737	1.158	1.070–1.253	** < 0.001**
Stage	I	Ref					
	II	1.784	1.702–1.870	** < 0.001**	3.899	3.599–4.224	** < 0.001**
	III	4.306	4.085–4.538	** < 0.001**	12.911	11.901–14.005	** < 0.001**
Histological type	IDC	Ref					
	ILC	1.015	0.949–1.084	0.672	1.009	0.918–1.109	1.009
	Others	0.941	0.867–1.021	0.143	0.894	0.794–1.008	0.894
HR status	ER + /PR +	Ref					
	ER + /PR-	1.508	1.420–1.601	** < 0.001**	2.163	1.993–2.348	** < 0.001**
	ER-/PR +	2.036	1.721–2.409	** < 0.001**	3.435	2.798–4.217	** < 0.001**
	ER-/PR-	2.020	1.922–2.124	** < 0.001**	3.419	3.201–3.652	** < 0.001**

*Abbreviation*: *HR* Hormone receptor, *ER* Estrogen receptor, *PR* Progesterone receptor, *HER-2* Human epidermal growth factor receptor 2, *IDC* Invasive ductal carcinoma, *ILC* Invasive lobular carcinoma, *BCS* Breast conserving surgery, *M* Mastectomy, *BCSS* Breast cancer specific survival, *CI* Confidence interval, *OS* Overall survival. Bold values indicate that they are statistically significant at *P* ≤ 0.05

**Table 4:** Multivariate analysis of the OS and the BCSS in Cohort 1

Patient characteristic		OS			BCSS		
		Hazard Ratio	CI (95%)	*P*	Hazard Ratio	CI (95%)	*P*
Age at diagnosis	20–30	Ref			Ref		
	31–40	0.876	0.672–1.141	0.326	0.982	0.736–1.309	0.899
	41–50	0.699	0.542–0.903	**0.006**	0.748	0.566–0.989	**0.041**
	51–60	0.946	0.735–1.217	0.665	0.920	0.698–1.212	0.553
	61–70	1.343	1.045–1.726	**0.021**	0.942	0.714–1.243	0.674
	71–80	3.008	2.341–3.865	** < 0.001**	1.498	1.133–1.979	**0.004**
Race	White	Ref			Ref		
	Black	1.600	1.505–1.701	** < 0.001**	1.544	1.423–1.674	** < 0.001**
	Others	0.843	0.787–0.903	** < 0.001**	0.889	0.809–1.976	**0.014**
Histological grade	1	Ref			Ref		
	2	1.172	1.102–1.246	** < 0.001**	1.630	1.451–1.832	** < 0.001**
	3	1.540	1.439–1.648	** < 0.001**	2.682	2.377–3.025	** < 0.001**
T stage	1	Ref			Ref		
	2	1.606	1.476–1.747	** < 0.001**	1.697	1.521–1.894	** < 0.001**
	3	2.155	1.929–2.407	** < 0.001**	2.559	2.229–2.938	** < 0.001**
	4	3.614	3.159–4.135	** < 0.001**	4.497	3.831–5.279	** < 0.001**
N stage	0	Ref			Ref		
	1	1.413	1.319–1.514	** < 0.001**	1.679	1.534–1.838	** < 0.001**
	2	1.974	1.730–2.252	** < 0.001**	2.550	2.179–2.984	** < 0.001**
	3	2.867	2.514–3.369	** < 0.001**	3.870	3.315–4.517	** < 0.001**
HER-2	Negative	-			Ref		
	Positive	-	-	**-**	0.599	0.553–0.650	** < 0.001**
Stage	I	Ref			Ref		
	II	1.005	0.910–1.109	0.925	1.574	1.368–1.811	** < 0.001**
	III	1.163	0.987–1.370	0.072	1.928	1.564–2.377	** < 0.001**
HR status	ER + /PR +	Ref			Ref		
	ER + /PR-	1.247	1.173–1.325	** < 0.001**	1.686	1.550–1.834	** < 0.001**
	ER-/PR +	1.567	1.321–1.858	** < 0.001**	2.090	1.696–2.574	** < 0.001**
	ER-/PR-	1.501	1.417–1.589	** < 0.001**	2.019	1.872–2.178	** < 0.001**

*Abbreviation*: *HR* Hormone receptor, *ER* Estrogen receptor, *PR* Progesterone receptor, *HER-2* Human epidermal growth factor receptor 2, *IDC* Invasive ductal carcinoma, *ILC* Invasive lobular carcinoma, *BCS* Breast conserving surgery, *M* Mastectomy, *BCSS* Breast cancer specific survival, *CI* Confidence interval, *OS* Overall survival. Bold values indicate that they are statistically significant at *P* ≤ 0.05

### Association of clinical factors with pCR in Cohort 2

Cohort 2 were NAC patients from Harbin Medical University Cancer Hospital, where 144 (16.4%) of the entire cohort achieved pCR, and 735 (83.6%) did not achieve pCR. The univariate analysis determined the factors affecting the pCR rate after NAC. T stage, ER expression, PR expression, HER-2 expression, KI67 expression, histological grade, HR status, and clinical stage were closely related to the pCR rate (*P* < 0.05) (Table 5). However, there was no significant correlation between age, chemotherapy regimen, N stage, surgical method, menopausal status, BMI, P53 expression, and pCR (*P* > 0.05). Higher T stage, ER positive, PR positive, HER-2 negative, and higher histological grade made patients less likely to achieve pCR. One individual (9.1%) of the ER-/PR + phenotype reached pCR, the ER-/PR- phenotype most easily achieved pCR (32.0%), and the lowest pCR rate for the ER + /PR + phenotype (8.3%). Including statistically significant factors in univariate analysis (excluded because large differences in histological grade and pathology type influenced the results), Logistic regression showed that HER-2 positive patients were more likely to achieve pCR than negative patients (OR = 2.057, CI 95% 1.389–3.046, *P* < 0.001). The high KI67 expression group was likelier to achieve pCR than the low expression group (OR = 1.777, CI 95% 1.47–2.754, *P* = 0.010). When the clinical stage was III, the odds of achieving pCR increased (OR = 1.682, CI 95% 1.075–2.631, *P* = 0.023). In this cohort, the ER + /PR- phenotype (OR = 1.949, CI 95% 1.037–3.663, *P* = 0.038) and ER-/PR- phenotype (OR = 2.697, CI95% 1.695–4.292, *P* < 0.001) achieved pCR more easily than patients with the ER + /PR + phenotype, while not statistically significant between the ER-/PR + phenotype and ER + /PR + phenotype (Table 6).

**Table 5:** Univariate analysis between clinical characteristics and pCR in Cohort2

Patient characteristic		All patients				*χ2*	*P*
		pCR(n = 144)		NpCR(n = 735)			
		N	%	N	%		
Age	≤ 40	30	20.8	130	17.7	0.801	0.371
	> 40	114	79.2	605	82.3		
Surgical methods	BCS	4	27.8	32	4.4	0.761	0.383
	M	140	72.2	703	95.6		
Menstruatio	Yes	72	50.0	387	52.7	0.340	0.560
	No	72	50.0	348	47.3		
BMI	≤ 18.5	1	0.6	18	2.4	3.488	0.322
	18.5–24	73	50.7	325	44.2		
	24–30	61	42.4	348	47.3		
	≥ 30	9	6.3	44	6.1		
Clinical T stage	1	31	21.5	76	10.3	**18.043**	** < 0.001**
	2	101	70.1	536	73.0		
	3	12	8.4	123	16.7		
Clinical N stage	0	26	18.1	84	11.4	5.099	0.165
	1	79	54.9	420	57.1		
	2	14	9.7	86	11.7		
	3	25	17.3	145	19.8		
ER	Positive	50	34.7	434	59.0	**28.795**	** < 0.001**
	Negative	94	65.3	301	41.0		
PR	Positive	32	22.2	350	47.6	**31.607**	** < 0.001**
	Negative	112	77.8	385	52.4		
HER-2	Positive	84	58.3	250	34.0	**31.139**	** < 0.001**
	Negative	60	41.7	485	64.0		
KI67	≤ 15	32	22.2	267	36.3	**10.672**	** < 0.001**
	> 15	112	77.8	468	63.7		
HR status	ER + /PR +	31	21.5	340	46.3	**35.105**	** < 0.001**
	ER + /PR-	19	13.2	94	12.8		
	ER-/PR +	1	0.7	10	1.4		
	ER-/PR-	93	64.6	291	39.5		
P53	0	66	45.8	395	53.8	6.215	0.102
	1	34	23.6	175	23.8		
	2	17	11.8	79	10.7		
	3	27	19.8	86	11.7		
Histological type	IDC	0	0	583	79.3	**418.91**	** < 0.001**
	ILC	0	0	21	2.9		
	DCIS	30	20.8	3	0.4		
	Others	114	79.2	128	17.4		
Histological grade	0–1	144	100	183	12.5	**290.70**	** < 0.001**
	2	0	0	451	61.4		
	3	0	0	101	26.1		
Stage	I	0	0	13	1.8	**10.105**	**0.006**
	II	103	71.5	429	58.4		
	III	41	28.5	293	39.8		

*Abbreviation*: *pCR* Pathologic complete response, *NpCR* Non-pathologic complete response, *HR* Hormone receptor, *ER* Estrogen receptor, *PR* Progesterone receptor, *HER-2* Human epidermal growth factor receptor 2, *IDC* Invasive ductal carcinoma, *ILC* Invasive lobular carcinoma, *DCIS* Ductal carcinoma in situ, *BCS* Breast conserving surgery, *M* Mastectomy. Bold values indicate that they are statistically significant at *P* ≤ 0.05

**Table 6:** Multivariate analysis between clinical characteristics and pCR in Cohort2

Patient characteristic		B	S. E	Wals	OR	CI (95%)	*P*
Clinical T stage	3	Ref					
	1 + 2	0.668	0.355	3.543	1.950	0.973–3.909	0.060
HR status	ER + /PR +	Ref					
	ER + /PR-	0.667	0.322	4.292	1.949	1.037–3.663	**0.038**
	ER-/PR +	0.039	1.083	0.001	0.962	0.115–8.042	0.977
	ER-/PR-	0.992	0.237	17.523	2.697	1.695–4.292	** < 0.001**
HER-2	Negative	Ref					
	Positive	0.721	0.200	12.956	2.057	1.389–3.046	** < 0.001**
KI67	≤ 15	Ref					
	> 15	0.575	0.224	6.615	1.777	1.147–2.754	**0.010**
Stage	I + II	Ref					
	III	0.520	0.321	52.415	1.682	1.075–2.631	**0.023**

*Abbreviation*: *HR* Hormone receptor, *ER* Estrogen receptor, *PR* Progesterone receptor, *HER-2* Human epidermal growth factor receptor 2, *CI* Confidence interval, *OR* Odds ratio. Bold values indicate that they are statistically significant at *P* ≤ 0.05

### Describe the patient's pCR according to the RECIST standard

**Fig. 9:**
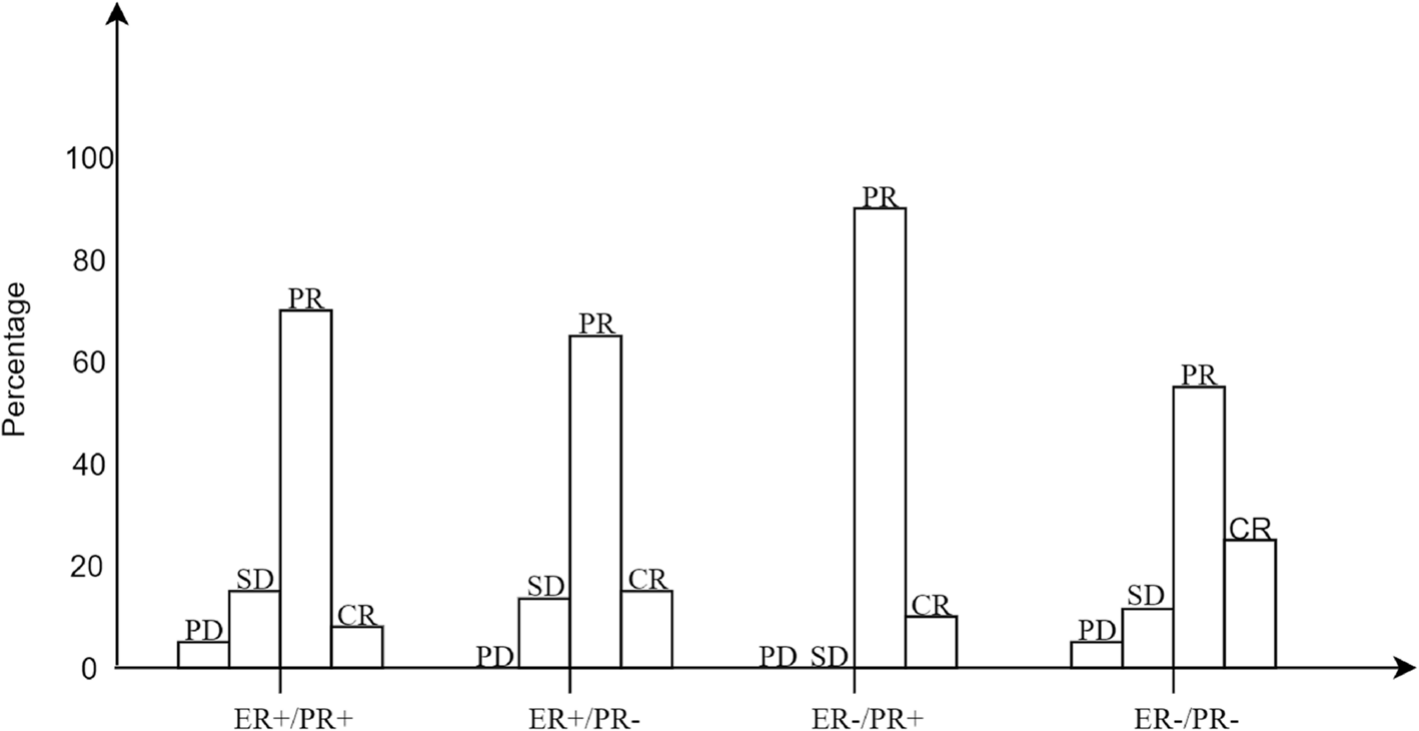
Group differences between the HR status using RECIST as the pathological criteria Abbreviation: ER, estrogen receptor; PR, progesterone receptor; PR, partial response; CR, complete response; PD, progressive disease, SD, stable disease

Clinical efficacy was evaluated according to the efficacy evaluation criteria of the Response Evaluation Criteria in Solid Tumors (RECIST) version 1.1. Partial response (PR) and complete response (CR) were defined as excellent clinical responses; progressive disease (PD) and stable disease (SD) were defined as poor clinical responses. In cohort 2, there was a significant relationship between the chemotherapy effect and different HR statuses, and 155 (17.5%) patients were insensitive to chemotherapy, and the overall chemotherapy effect was good. In patients with the ER-/PR + phenotype without PD and SD, 10 (90.9%) patients achieved PR, and 1 (9.1%) patient achieved CR. Patients with the ER + /PR + phenotype had the least effective chemotherapy, with 75 (20.2%) patients having no significant change in the mass. The chemotherapy effects of the ER + /PR- and ER-/PR- phenotypes are similar (Fig. 9).

## Discussion

This study discussed the biological significance, sensitivity to chemotherapy, and survival outcome of the ER-/PR + phenotype BC. HR status is an independent factor affecting the prognosis and chemotherapy effect of patients with BC. In these two cohorts, 704 (0.9%) and 11 (1.3%) patients had ER-/PR + phenotypes, respectively. This is consistent with the results of previous studies, Keshgegian found that ER-/PR + phenotype BC accounted for 1.5% of all cases but also proved that ER-/PR + phenotype is an objective existence of a rare subtype [15, 16]. We found that the ER-/PR + phenotype often appears in pre-menopausal women, most of which are typical IDC. Rhodes conducted a giant experiment in which he analyzed data from 7016 cases of BC in 71 laboratories and found that ER-/PR + phenotypes occurred at a high frequency under the age of 50 [17]. In addition, p53 and other biomarkers are relatively high in patients with this phenotype. Kunc found that about 8% of patients showed the ER-/PR + phenotype, and these women were almost the same age as the ER-/PR- phenotype. These patients had higher lymph node metastasis rates and histological grades, and HER-2 was often expressed as positive [18].

In 2004, Olivotto believed that PR was meaningless in BC treatment decisions and only needed to consider the expression of ER because nearly 100% of BC patients in their cohort had ER expression consistent with PR expression and should stop PR testing in BC therapy [19]. However, many experts soon denied this view because experts found that when the expression of PR differs, the survival outcome will change. This effect is more substantial than ER, proving that PR is an independent prognostic factor for patients with BC and that ER-/PR + and ER + /PR- phenotypes are two different BC, which need to be distinguished [20, 21]. There are many factors for the existence of the ER-/PR + phenotype. Fuqua found that some ER mutations lead to the lack of exon 5 of the hormone-binding domain, which can still stimulate the reactive expression of PR when ER is negative, which can be explained as a potential biological mechanism of the ER-/PR + phenotype [22]. Moreover, Onitilo found that young women had high estrogen levels. The ER was saturated when the estrogen content increased, preventing the ligand's ER from binding to the lesion and reducing the expression [23].

Some experts believe that the emergence of the ER-/PR + phenotype is not caused by biological factors but by technical reasons. When the surgeon is in surgery, the cauterization of the electric knife can lead to overheating of the tissue, which can also lead to false negative results. Allred recommends that when this classification occurs, it needs to be re-evaluated by a specialized pathologist [24]. Nadji evaluated 5993 cases of primary invasive BC with IHC and found that the ER-/PR + phenotype did not exist, which was caused by irregular operation of IHC [25]. De Maeyer also re-evaluated the IHC of 32 BC patients with the ER-/PR + phenotype in local hospitals and found that all patients were ER + /PR + phenotype [26]. Apple suggested that different fixative agents and fixation times would affect the accuracy of ER and PR [27]. Collins studied the IHC results of 825 BC patients and found that BC patients had either ER + /PR + phenotype or ER-/PR- phenotype, and there were no weakly stained cases [28]. When Goldstein detects the expression of ER, ER may be washed away during the dehydration step, resulting in a decrease or even negative expression of ER [29]. Therefore, we should understand that the IHC process, ER, and PR antibodies applied in each hospital differ, and pathologists' experience in viewing slices is also different. It is unrealistic to achieve standardized and unified results in the world.

In addition to the technical errors in IHC, the change of diagnostic threshold will also affect the appearance of the ER-/PR + phenotype, and the positive cutoff point of the international committee has been changing in recent decades. In 2000, the National Institutes of Health thought any obvious staining should be counted as a positive result, while some people thought that 10%, 20%, or even 50% nuclear staining could be considered a positive result [30]. Yamashita believes that cells with low expression of ER or low expression of PR have a better survival rate after recurrence. It is recommended that the cutoff value be set lower, such as 1%, especially for metastatic diseases [31].

Dabbs was evaluated using the best-fixed tissue method and found that 5% of BC tumors still had ER-/PR + phenotype, proving that the ER-/PR + phenotype was actual and not artificial [32]. After Itoh re-evaluated the tumor tissue using gene expression profiles, 25% of the cases had the same HR phenotype. They again denied the view that the ER-/PR + phenotype was a technical artifact [33]. Ahmed used a Tissue Micro-Array (TMA) to re-evaluate 267 BC patients with the ER-/PR + phenotype, and 92 patients were still defined as ER-/PR + phenotype, which may be because traditional IHC usually can only label one or two antigens in tissues. Quantitative interpretation often lacks an objective standard [34]. Borras established the Evsa-T clonal cell prediction model, essential for discovering and evaluating the ER-/PR + phenotype [35]. Schroth analyzed PAM50 expression characteristics and pathways from The Cancer Genome Atlas (TCGA) BC data sets to test different molecular characteristics and proved ER-/PR + phenotype [36]. The impact of false positive results on the treatment of BC patients is enormous, and in cancer patients with high expression of PR, at least some BC cannot detect the expression of ER [37]. Therefore, we should give priority to improving the quality of IHC testing methods, and we need to ensure that all laboratories that conduct IHC testing for HR in BC follow other quality control and assurance measures outlined in the forthcoming guidelines of the American Society of Clinical Oncology and the American College of Pathologists [38]. Regardless of the diagnostic needle and sample preparation process, we may find BC patients with the ER-/PR + phenotype in routine practice.

The tumor has heterogeneous characteristics, and experts have found that the expression of ER and PR plays a vital role in guiding clinical treatment and predicting survival outcomes. The treatment is easy to find in the case of HR double positive or double negative. Double-positive patients can reduce tumor invasiveness, prevent recurrence, and prolong life through endocrine therapy. Double-negative patients are likely higher-grade tumors closely related to higher recurrence rates, lower OS, and anti-endocrine therapy. Although there are more adverse reactions to chemotherapy, such patients can gain more survival benefits from systemic chemotherapy [39]. Ng believes tamoxifen adjuvant hormone therapy has the same survival advantage in patients with ER + /PR + and ER-/PR + phenotypes but has little effect on ER + /PR- phenotypes. Patients with the ER-/PR + phenotype are more aggressive, and the survival rate of patients with the ER + /PR + phenotype is similar to that of patients with the ER + /PR + phenotype [40]. Ethier also found that the ER-/PR + phenotype was similar to the ER + /PR + phenotype regarding molecular subtype and outcome [41]. Rakha came to the opposite conclusion. He found that patients with the ER + /PR + phenotype had a better prognosis than those with the ER-/PR + phenotype [42]. Davies also found an essential difference between the ER + /PR + and ER-/PR + phenotypes. Patients with ER + /PR + phenotype had a good prognosis, while patients with ER-/PR + phenotype could not benefit significantly from endocrine therapy [43].

Therefore, when we use endocrine therapy for such patients, we should be careful not to put endocrine therapy in the first place [44]. This study found that the ER-/PR + phenotype showed different clinicopathological characteristics and survival outcomes compared with other phenotypes. Compared with patients with ER + /PR + phenotype, patients with ER-/PR + phenotype showed a worsening OS and BCSS. The results were similar to the ER-/PR- phenotype and compared with other phenotypes. ER-/PR + phenotype was more likely to reach pCR. The mechanism of high sensitivity to chemotherapy is not precise. Some experts speculate this may be due to insufficient chemotherapy in patients with this phenotype. Zheng found that the risk of death of the ER-/PR + phenotype was the highest of all phenotypes in the first 1–2 years and then decreased rapidly during 3–5-year follow-up. Therefore, it is recommended that patients receiving chemotherapy at the early stage of the disease can significantly reduce the risk of death [45].

Our study still has many limitations. First, the center of our hospital is NAC patients, and we cannot observe the same survival results as the SEER database. In the future, we must conduct a long-term postoperative follow-up to study the relationship between different HR statuses and OS and BCSS. Secondly, this study is a retrospective analysis, which may need to be more convincing because the incidence of BC patients with ER-/PR + phenotype is very low, so it is still difficult to conduct a prospective study. Prospective studies on the response of the ER-/PR + phenotype BC to endocrine therapy can be carried out in the future.

## Conclusion

In conclusion, different HR statuses are independent factors affecting BC patients' chemotherapy effect and prognosis. The ER-/PR + phenotype is a specific BC subtype with unique clinicopathological features and prognosis. BC patients with the ER-/ PR + phenotype have a higher sensitivity to chemotherapy and a prognosis intermediate between the ER + /PR + and ER-/PR- phenotype, preferring the ER-/PR- phenotype. It should not be treated as conventional-type Luminal tumors but can be treated as per HER-2 expression. We need to pay more attention to this part of the group, and the government should develop additional policies to help patients achieve precise individualized treatment.

## Data Availability

The data and materials for this study are authentic and available. All the data can be found in the digital integrated management system of Harbin Medical University Cancer Hospital. Publicly archived datasets analysed or generated during the study may be provided by the corresponding author upon reasonable request. The datasets generated during and/or analysed during the current study are available from the corresponding author on reasonable request.

## Abbreviations

ALND: Axillary lymph node dissection
ASCO: American Society of Clinical Oncology
AJCC: American Joint Committee on Cancer
BC: Breast cancer
BCS: Breast conserving surgery
BCSS: Breast cancer specific survival
BMI: Body mass index
CI: Confidence interval
DCIS: Ductal carcinoma in situ
ER: Estrogen receptor
HER-2: Human epidermal growth factor receptor 2
HR: Hormone receptor
IDC: Invasive ductal carcinoma
IHC: Immunohistochemical
ILC: Invasive lobular carcinoma
NAC: Neoadjuvant chemotherapy
OS: Overall survival
PR: Progesterone receptor
pCR: Pathologic complete response
PSM: Propensity score matching
RECIST: Response Evaluation Criteria in Solid Tumors
ROC: Receiver operating characteristic
SEER: Surveillance, Epidemiology, and End Results
SLNB: Sentinel lymph node biopsy
TCGA: The Cancer Genome Atlas
TMA: Tissue Micro-Array
